# A Bifunctional Electrocatalyst for Oxygen Evolution and Oxygen Reduction Reactions in Water

**DOI:** 10.1002/ange.201508404

**Published:** 2016-01-15

**Authors:** Wolfgang Schöfberger, Felix Faschinger, Samir Chattopadhyay, Snehadri Bhakta, Biswajit Mondal, Johannes A. A. W. Elemans, Stefan Müllegger, Stefano Tebi, Reinhold Koch, Florian Klappenberger, Mateusz Paszkiewicz, Johannes V. Barth, Eva Rauls, Hazem Aldahhak, Wolf Gero Schmidt, Abhishek Dey

**Affiliations:** ^1^ Institute of Organic ChemistryJohannes Kepler University LinzAltenberger Strasse 694040LinzAustria; ^2^ Department of Inorganic ChemistryIndian Association for the Cultivation of Science2A & 2B Raja SC Mullik RoadKolkata700032India; ^3^ Radboud UniversityInstitute for Molecules and MaterialsHeyendaalseweg 1356525AJ NijmegenThe Netherlands; ^4^ Institute of Semiconductor and Solid State PhysicsJohannes Kepler University LinzAltenberger Strasse 694040LinzAustria; ^5^ Physics Department E20Technische Universität MünchenJames-Franck-Strasse 185748GarchingGermany; ^6^ Department of PhysicsPaderborn UniversityWarburger Strasse 10033098PaderbornGermany

**Keywords:** Corrole, Elektrochemie, Mangan, Sauerstoffentwicklung, Sauerstoffreduktion

## Abstract

Oxygen reduction and water oxidation are two key processes in fuel cell applications. The oxidation of water to dioxygen is a 4 H^+^/4 e^−^ process, while oxygen can be fully reduced to water by a 4 e^−^/4 H^+^ process or partially reduced by fewer electrons to reactive oxygen species such as H_2_O_2_ and O_2_^−^. We demonstrate that a novel manganese corrole complex behaves as a bifunctional catalyst for both the electrocatalytic generation of dioxygen as well as the reduction of dioxygen in aqueous media. Furthermore, our combined kinetic, spectroscopic, and electrochemical study of manganese corroles adsorbed on different electrode materials (down to a submolecular level) reveals mechanistic details of the oxygen evolution and reduction processes.

## Graphical abstract

**Doppelleben**: Ein Mangan‐Corrol‐Komplex vermittelt als difunktioneller Katalysator in wässriger Lösung die elektrokatalytische Erzeugung von Disauerstoff ebenso wie seine Reduktion. In schwach bis mäßig alkalischem Medium oxidiert der Komplex Hydroxidionen über einen Vier‐Elektronen‐Prozess zu molekularem Sauerstoff, und er reduziert O_2_ über einen Zwei‐Elektronen‐Prozess zu Wasserstoffperoxid.

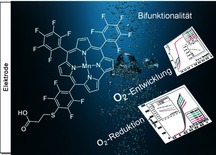

In nature, the oxidation of water is catalyzed by the Mn_4_Ca inorganic unit embedded in the D1 protein subunit of photosystem II (PSII).^1^ The water oxidation complex of PSII has inspired a wide range of model molecular water oxidation catalysts (WOCs). As in the WOC of PSII, high oxidation states of the metals are generally stabilized by introducing electron‐donating ligands with oxygen and nitrogen donor atoms.^2^ The development of synthetic catalysts which are able to mediate water oxidation and dioxygen reduction under mild conditions with a minimized energy demand has become an appealing challenge.^3^ Recently, many different examples of these molecular catalysts were described in detail by Åkermark and co‐workers.^4^ Homogeneous and heterogeneous molecular catalysts offer attractive features, such as controllable redox properties, ease of mechanistic investigation, and available strategies for the characterization of reactive intermediates.^5^ A variety of homogeneous and heterogeneous water‐oxidation catalysts based on transition metals have been developed, including complexes of Mn, Ru, Ir, and Co.^4^, ^6^ All of these catalyst systems are proposed to proceed through a high‐valent intermediate.^7^ Corroles are trianionic ligands known to stabilize metal ions in their high‐valent oxidation states.^8^ Nocera and co‐workers reported a hangman Co corrole for the efficient oxidation of water.^9^ Co corrole complexes are also reported for selective 4 e^−^/4 H^+^ oxygen reduction reactions.^10^ In contrast, Mn corroles are vastly unexplored as potential O_2_ evolution catalysts, although Mn is the metal responsible for natural water‐oxidation processes. The corrole macrocycle can stabilize the oxidation state of Co only as high as VI, but that of Mn up to VII. Moreover, the corrole macrocycle, in contrast to the closely related porphyrin‐based systems, tends to be involved as a non‐innocent ligand, forming π‐radical species.^11^ The stabilization of higher oxidation states determines the extraordinary properties of manganese(V)‐oxocorroles as oxygen atom transfer reagents for the epoxidation of alkenes^12^ or the O−O bond‐formation step in the artificial photosynthetic oxidation of water.^13^ Water oxidation is considered to be the bottleneck of the water splitting reaction.^14^ Hence, there is a strong motivation to develop catalysts that can oxidize water efficiently under ambient conditions. The development of efficient bifunctional catalysts has been a fast evolving area.^15^ Although there are some reports of bifunctional catalysts for the reversible conversion of protons into H_2_, reports on nonprecious metal based electrocatalysts which can efficiently oxidize water to oxygen and also reduce oxygen are rare. Such catalysts are in high demand.^16^ In the present study we have investigated the adsorption performance of manganese 5,10,15‐tris(pentafluorophenyl)corrole (MnTpFPC) analogues^17^ on different electrode materials in a thin‐film phase as well as down to the scale of individual molecules. We have developed a water‐soluble bifunctional electrocatalyst **1** with a single Mn site for the homogeneous and heterogeneous oxygen evolution reaction (OER) and homogeneous and heterogeneous oxygen reduction reaction (ORR) in aqueous solution (Figure 1).

**Figure 1:**
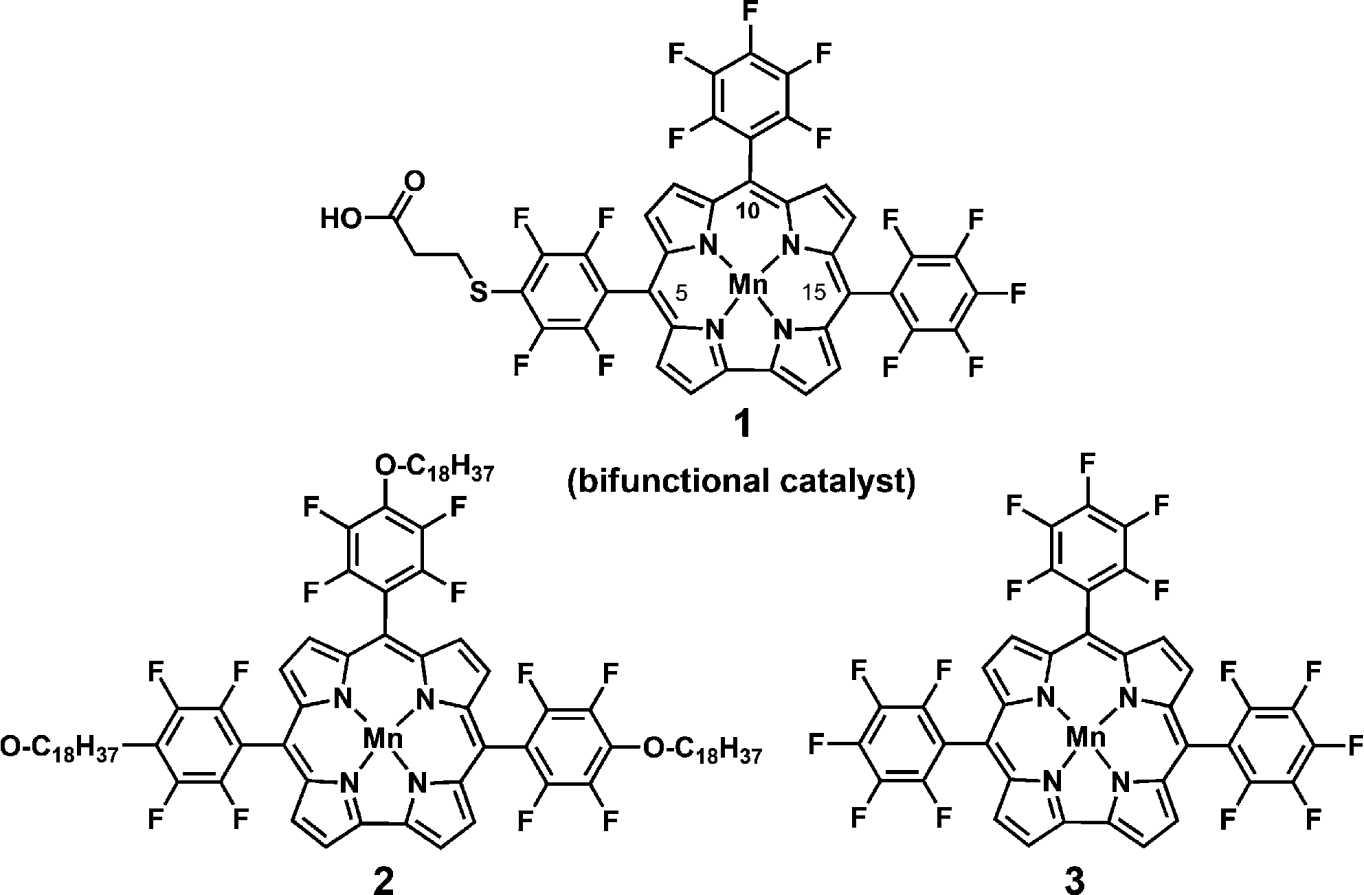
Water‐soluble bifunctional manganese corrole catalyst **1** (obtained as a mixture of regioisomers, see the Supporting Information) and reference compounds **2** and **3** for the study of the adsorption mode and the electronic properties of single molecules on solid surfaces.

Cyclic voltammetry (CV) studies of complex **1** in CH_3_CN solution shows two one‐electron oxidations corresponding to the Mn^III^/Mn^IV^ (0.53 V versus Ag/AgCl) and Mn^IV^/Mn^V^ (0.78 V versus Ag/AgCl) processes. There is an irreversible catalytic wave at a potential *E*_p,a_=1.38 V which appears following the oxidation of Mn^III^ to Mn^V^, thus suggesting the presence of an irreversible anodic process. The waveform for the Mn^IV^/Mn^V^ couple at *E*_1/2_=0.73 V is independent of the scan rate (ν) under these conditions. Its peak current (*i_p_*) varies linearly with ν^1/2^ (inset of Figure 2) and is consistent with diffusion‐limited electron transfer at the electrode.^18^

**Figure 2:**
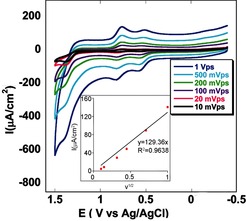
Cyclic voltammogram of catalyst **1** dissolved in acetonitrile on varying the scan rate (*ν*) from 10 mV s^−1^ to 1 V s^−1^ using glassy carbon as the working electrode. The inset shows the plot of the maximum catalytic current (*i*_p_) versus the scan rate (*ν* ^1/2^); linear fit (*y=*129.26 *x*, *R* ^2^=0.964).

The diffusion coefficient, *D*_cat_=2.32×10^−7^ cm^2^ s^−1^, was obtained from the scan‐rate dependence of *i*_p_ (inset of Figure 2) using the equation *i*_p_=0.4463 *n*_p_ *F* *A* *C*_cat._(*n*_p_ *FνD*/*R* *T*)^1/2^, where *i*_p_, *F*, *A*, *C*_cat._, *ν*, *D*, *R*, and *T* are the maximum noncatalytic current, Faraday constant, area of the electrode, bulk concentration of the catalyst, scan rate, diffusion coefficient of the catalyst in solution, ideal gas constant, and temperature, respectively.^19^ At slow scan rates, nearly ideal plateau wave shapes were observed, reaching a current maximum at 1.3 V. Reproducible CV measurements further indicate that the catalyst is stable following multiple catalytic turnovers.

The catalytic evolution of oxygen by **1** was investigated in acetonitrile solution containing 100 mm Bu_4_NClO_4_ (TBAP) by adding different concentrations of a base (NaOH in water; Figure 3 A). As the concentration of the base was gradually increased from 1 mm to 25 mm, a sharply increasing irreversible current was obtained. The plot of (*i*_c_/*i*_p_)^2^ versus the concentration of the base (Figure 3 B) shows a linear relationship, thus indicating that the process is pseudo‐first order with respect to the base. Evolution of O_2_ was confirmed by cathodic scans, where the O_2_ evolved in the anodic sweep was detected during the cathodic sweep (where it was reduced by **1**). Using the equation *I*_C_=*n* *F* *A* *C*_cat._ (*D* *k*_cat._)${}^{1/2}$ ,^20^ the pseudo‐first order rate constant (*k*_cat._) is calculated to be 11.4 s^−1^ at a concentration of 25 mm NaOH and a scan rate of 10 mV s^−1^. In this equation, *I*_c_ is defined as the maximum catalytic current and the other terms are as mentioned above.

**Figure 3:**
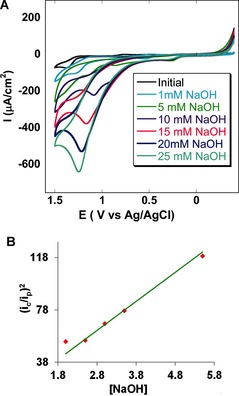
A) Cyclic voltammogram of catalyst **1** (in acetonitrile) showing homogeneously increasing catalytic oxygen evolution with increasing base concentration from 1 mm NaOH to 25 mm NaOH at a scan rate of 50 mV s^−1^. A glassy carbon electrode was used as the working electrode. B) Plot of (*i*_c_/*i*_p_)^2^ versus conc. of NaOH (in mm) in a homogeneous OER in acetonitrile at a scan rate of 100 mV s^−1^; linear fit (*y=*22.227 *x*, *R* ^2^=0.966)

With the aim of testing manganese corroles as heterogeneous catalysts, in the next step we investigated 1) the adsorption behavior and 2) the electronic properties of Mn corroles on different solid surfaces by cyclic voltammetry, scanning tunneling microscopy (STM), and spectroscopy. We selected substrate materials such as graphite and silver, which are highly relevant as electrode materials for heterogeneous catalysis. Moreover, we have studied **2** and **3** at the solid–liquid,^21^, ^22^ and the solid–vacuum interface—thus covering a wide range of catalytic interfaces for application. A prerequisite for STM studies under ultrahigh vacuum conditions is that the molecules can be carefully evaporated onto the substrate without any destruction of the molecules. We chose compound **3** for these experiments. Octadecyl chains need to be attached to the corrole macrocycle (compound **2**) to obtain proper orientation and adsorption for the STM experiments at the solid–liquid interface.^22^

Figure 4 A shows a typical STM image obtained at the solid–liquid interface of a self‐assembled monolayer of **2** on the basal plane of highly ordered pyrolytic graphite (HOPG). The monolayer was prepared by exposing the HOPG substrate to a 10 μm solution of **2** in 1‐phenyloctane at 295 K. In the image, lamellar arrays are visible in rotational domains of approximately 120°. In these lamellae, which have a periodicity of 4.4±0.2 nm, single molecules of **2** can be distinguished (marked by a red circle in Figure 4 A). The molecular layer clearly exhibits regions with diameters >20 nm and short‐range order, but it is rather disordered at longer distances. The individual molecules are imaged as elongated disklike shape features, thus suggesting their aromatic planes are oriented approximately parallel with respect to the surface. Occasionally, brighter features are observed with an apparent height of approximately twice that of the majority of the other molecules in the layer, which may indicate the presence of face‐to‐face stacked dimers. Repeated scanning of the same area did not result in detectable changes in the appearance of the molecular layer, thus indicating that the molecules remain stable at the solid–liquid interface.

**Figure 4:**
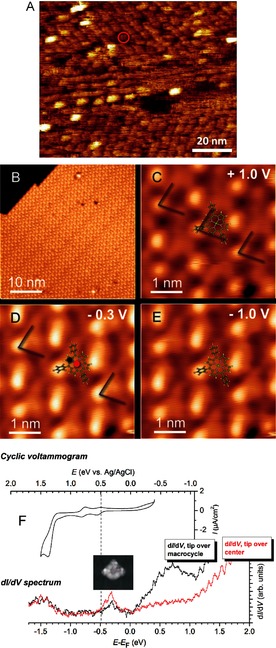
A) STM image of a self‐assembled monolayer of manganese corroles **2** at a solid–liquid interface on highly ordered pyrolytic graphite (HOPG) and 1‐phenyloctane. B–E) Low‐temperature STM images of ordered MnTpFPC (**3**) molecules on Ag(111) at 5 K. F) Nomogram comparing the cyclic voltammogram of **1** with the d*I*/d*V* spectrum of **3** observed by tunneling over the manganese corrole center (red spectrum) and the corrole macrocycle (black spectrum); insert: simulation of a STM image at a bias voltage of −0.3 V using the Tersoff–Hamann model (see also Figure S5).^26^

In contrast, Mn corroles were found to form highly ordered monolayer films at a solid–vacuum interface. The STM image of Figure 4 B shows a monolayer of manganese corroles **3** immobilized on a single‐crystal Ag(111) surface under ultrahigh vacuum at 5 K. A high degree of order is evident. Figure 4 C–E depict STM images recorded with increased magnification at different sample bias voltages. Compared to the results obtained at the solid–liquid interface, intramolecular features of the individual Mn corrole molecules within the layer are clearly resolved. As a guide to the eye, we have overlaid the structure model on the STM images to facilitate the assignment of the STM topography to different structural elements. The different structural elements of the manganese corrole molecule are discernible (metal center, pyrrole rings of the macrocycle, pentafluorophenyl substituents; see also Figure S5 and XPS data in Figure S6). Similar to our results on HOPG, we have found that manganese corrole molecules adsorb nondissociatively on Ag(111), with their macrocycles oriented approximately parallel to the substrate surface. The high spatial resolution and exceptional drift stability obtained at the solid–vacuum interface at low temperatures (Figures 4 B–E) facilitates local spectroscopy of the frontier orbital electronic structure of different functional groups on single Mn corrole molecules within the layer. Figure 4 F (bottom) shows typical tunneling conductance spectra obtained with the STM tip fixed at a constant height over the macrocycle (black curve) and the Mn center (red curve) of single manganese corrole molecules **3** adsorbed on Ag(111).

For comparison, the top part of Figure 4 F shows the cyclic voltammogram of **1** in solution. Notice that the energy axes of both methods are related and, thus, can be interconverted. For a proper comparison, the energy values from the two methods have to be referred to a common zero level, for example, as represented by the common vacuum level. A well‐established referencing procedure has been described by Mazur et al.^23^ The interconversion between different reference cathodes is explained in Ref. 24; the work function of Ag(111) is 4.74 eV.^25^

A detailed analysis reveals that the half‐wave potential energies of the first oxidation (observed by cyclovoltammetry, upper curve) lie close to the onset of the broad d*I*/d*V* peak, which originates from tunneling out of occupied metal‐centered molecular orbitals lying close below the Fermi level of Ag(111) (lower red curve and simulated STM image in Figure 4 F). This finding indicates that the active center of the surface‐immobilized catalyst exists as Mn^III^ and, most probably, exhibits similar catalytic properties as the dissolved catalyst in solution. This is a promising result in view of expanding the potential applications of manganese corroles towards heterogeneous catalysis at the solid–gas interface.

Indeed, catalytic oxygen evolution was confirmed by varying the pH value of the buffer (Figure 5) after immobilizing the catalyst **1** on an edge plane pyrolytic graphite (EPG) electrode. The sample was purged with argon before each scan to remove O_2_. Only instantly generated O_2_ during oxidative scans was detected on reverse scans at −0.3 V versus NHE (peak potential (*E*_p_) for the O_2_/O_2_^−^ couple). At pH 7.0, very slow OER kinetics were detected at potentials >1.3 V. However, as the pH value of the buffer was increased, enhanced electrocatalytic OER currents were observed, and at pH 11.0 high, mass transfer limited, catalytic current was observed. **1** is capable of reducing the O_2_ which was produced during the OER (see Figure 5, inset). The generated electrocatalytic current increases at a potential of about −0.35 V. Evidence of oxygen evolution also resulted from rotating ring‐disk electrochemistry (RRDE, Figure 6 A) studies on physisorbed catalyst **1** on an edge plane graphite (EPG) electrode under anaerobic conditions, with the platinum at a constant potential of 0.3 V. The generated oxygen was reduced by platinum in situ. The RRDE experiments suggest a normal substrate diffusion limited current. The hydroxide oxidation current increases in accordance with the Koutecky–Levich Equation discussed below. A linear plot was observed when *i* ^−1^ was plotted against the inverse square root of the angular rotation rate (*ω* ^−1/2^; inset of Figure 6 B). From the intercept of this plot, the second order rate constant was determined to be 1.55×10^4^ m ^−1^ s^−1^. During the process of oxygen evolution, RRDE experiments were performed to detect whether any partially oxidized species were generated. To do so, a Pt ring was kept at a constant potential of 0.2 V, where Pt can oxidize the generated partially oxidized species, such as H_2_O_2_ and O_2_^−^. From the RRDE data we could determine the generation of partially oxidized species at a yield as low as 2.1 % (Figure S7). To calculate the turnover number (TON) and the turnover frequency (TOF), we physisorbed the catalyst on an edge plane graphite electrode and performed controlled potential electrolysis at a potential of 1.4 V versus Ag/AgCl for 11.1 h.

**Figure 5:**
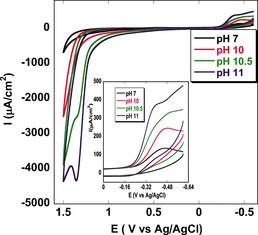
Anaerobic cyclic voltammogram of **1** immobilized on an edge plane pyrolytic graphite (EPG) electrode on varying the pH value from pH 7.0 to pH 11.0, which indicates the bifunctional nature of the catalyst. The inset shows the zoomed portion of the ORR where produced oxygen during the OER gets reduced.

**Figure 6:**
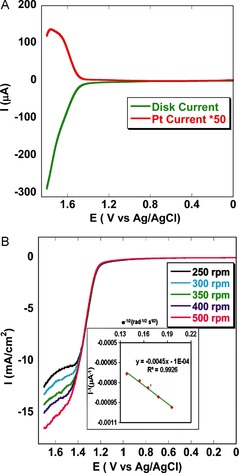
A) RRDE data of **1** physisorbed on an EPG electrode in pH 11.0 buffer at a constant rotation of 300 rpm and scan rate of 10 mV s^−1^, with platinum held at a constant potential of 0.3 V where it reduced the oxygen generated during the OER (Figure S8). B) Linear sweep voltammograms of immobilized catalyst on an EPG electrode at a scan rate of 50 mV s^−1^ on varying the rotation rate from 250 to 500 rpm. The inset shows the Koutecky–Levich plot (*I* ^−1^ versus *ν* ^−1/2^) from which *k*_cat._ was calculated; linear fit (*y*=−0.0045 *x*, *R* ^2^=0.993).

A TON of 1.90×10^4^ over 11.1 h was obtained, thus illustrating that the catalyst is highly stable at the electrode surface. The TOF was calculated to be 0.47 s^−1^ (Figure S9). Finally, we measured the generated oxygen by means of an inverted burette technique. A Faradaic efficiency of 82 % was calculated (Figure S10).

When the catalyst was physisorbed on an edge plane graphite electrode in the absence of oxygen at pH 7.0, a reversible one‐electron reduction was conducted at *E*°_1/2_≈−0.25 V (versus Ag/AgCl; Figure 7 A). In air‐saturated buffer, a large irreversible oxygen reduction current superseded the reversible process. The onset potential of this large reduction current is responsible for the catalytic reduction of oxygen. A second electrocatalytic ORR was observed at −0.6 V, which also catalyzes the ORR process. A normal substrate diffusion limited current was observed at potentials below −0.6 V when rotating disk electrochemistry (RDE; Figure 7 B) was performed. When the rotation rate was increased, the O_2_ reduction current increased according to the Koutecky–Levich Equation, *i* ^−1^=*i*_K_(E)^−1^1 + *i*_L_^−1^, where *i*_K_(E) is the potential‐dependent kinetic current and *i*_L_ is the Levich current. *i*_K_(E) and *i*_L_ are defined as *n* *F* *A*[O_2_]*k*_cat._ *Γ*_catalyst_ and 0.62 *n* *F* *A*[O_2_](*D* ${}_{O{}_{2}}$ )^2/3^ *ω* ^1/2^ *ν* ^−1/6^, respectively, where *n* is the number of electrons transferred to the substrate, *A* is the macroscopic area of the disk (0.096 cm^2^), [O_2_] is the concentration of O_2_ in an air‐saturated buffer (0.26 mm) at 25 °C, *k*_cat._ is the second order rate constant of the electrocatalytic reduction of O_2_, *Γ*_catalyst_ is the catalyst concentration in moles cm^−3^, *D* ${}_{O{}_{2}}$ is the diffusion coefficient of O_2_ (1.9×10^−5^ cm^2^ s^−1^) at 25 °C, *ω* is the angular velocity of the disc, and ν is the kinematic viscosity of the solution (0.009 cm^2^ s^−1^) at 25 °C.^19^

**Figure 7:**
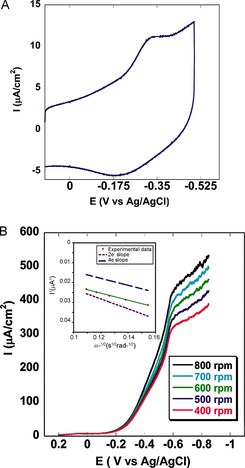
A) Cyclic voltammogram (anaerobic) of the catalyst **1** in pH 7.0 buffer on an edge plane graphite electrode. B) Linear sweep voltammogram of **1** physiadsorbed on an EPG electrode in pH 7.0 buffer at a scan rate of 50 mV s^−1^ with different rotation rates. The inset shows the Koutecky–Levich plot of the catalyst showing the ORR. The dotted and dashed lines are used to denote the theoretical plots for 2 e^−^ and 4 e^−^, respectively.

A linear plot was observed when *i* ^−1^ values at multiple rotation rates were plotted against the inverse square root of the angular rotation rate (*ω* ^−1/2^; inset of Figure 7 B). The number of electrons (*n*) involved in the O_2_ reduction by a catalytic species may be calculated from the slope, and the second order rate of catalysis (*k*_cat._) is obtained from the intercept of this linear plot. The slopes obtained from the experimental data at different potentials in the substrate diffusion controlled region are consistent with the theoretical slope presumed for a 2.3 e^−^ process. This is in accordance with the RRDE data, which shows approximately 90 % partially reduced oxygen species (PROS) and is consistent with previous reports on manganese corroles.^13^, ^27^ The intercept of the Koutecky–Levich plot can be used to evaluate the *k*_cat._ value (*i*_K_=*n* *F* *A*[O_2_]*k*_cat._ *Γ*_catalyst_). At −0.64 V (versus Ag/AgCl), the rate of the 2 e^−^ reduction of O_2_ is 3.81×10^3^ m ^−1^ s^−1^.

To conclude, the manganese corrole complex **1** exhibits bifunctional character in aqueous solution by oxidizing hydroxide ions through a four‐electron process in weak to moderate alkaline conditions to molecular oxygen and reducing O_2_ in a two‐electron process to hydrogen peroxide. The complex can produce a steady OER current with a faradaic efficiency of >80 % over 11 h. The selectivity, fast kinetics, and stability of this complex suggest that manganese corroles should definitely be considered as potential candidates for bifunctional catalysts for the reversible conversion of oxygen into water. Our combined STM results—obtained at the solid–liquid and solid–vacuum interface—have revealed 1) the nondissociative and regular adsorption of manganese corrole molecules on electrode surfaces and 2) intact electronic properties of manganese corroles. Both results are crucial for heterogeneous catalysis at the solid–liquid as well as solid–gas interfaces.

## Supporting information

As a service to our authors and readers, this journal provides supporting information supplied by the authors. Such materials are peer reviewed and may be re‐organized for online delivery, but are not copy‐edited or typeset. Technical support issues arising from supporting information (other than missing files) should be addressed to the authors.

Supplementary
